# PILER-CR: Fast and accurate identification of CRISPR repeats

**DOI:** 10.1186/1471-2105-8-18

**Published:** 2007-01-20

**Authors:** Robert C Edgar

**Affiliations:** 1 45 Monterey Dr., Tiburon, CA, USA

## Abstract

**Background:**

Sequencing of prokaryotic genomes has recently revealed the presence of CRISPR elements: short, highly conserved repeats separated by unique sequences of similar length. The distinctive sequence signature of CRISPR repeats can be found using general-purpose repeat- or pattern-finding software tools. However, the output of such tools is not always ideal for studying these repeats, and significant effort is sometimes needed to build additional tools and perform manual analysis of the output.

**Results:**

We present PILER-CR, a program specifically designed for the identification and analysis of CRISPR repeats. The program executes rapidly, completing a 5 Mb genome in around 5 seconds on a current desktop computer. We validate the algorithm by manual curation and by comparison with published surveys of these repeats, finding that PILER-CR has both high sensitivity and high specificity. We also present a catalogue of putative CRISPR repeats identified in a comprehensive analysis of 346 prokaryotic genomes.

**Conclusion:**

PILER-CR is a useful tool for rapid identification and classification of CRISPR repeats. The software is donated to the public domain. Source code and a Linux binary are freely available at .

## Background

**Figure 1:**
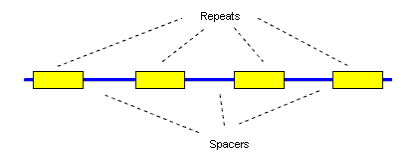
**Structure of a CRISPR array**. CRISPR repeats are perfectly (or almost perfectly) conserved short sequences, typically of length 20 to 40 bases, separated by unique sequences known as spacers. The spacer length in a given array is sometimes approximately conserved, varying by a few bases, and sometimes exactly conserved. The spacer length is typically similar to the repeat length.

Recent analysis of prokaryotic genomes has lead to the discovery of a new class of repeat characterized by short, perfectly conserved elements of typical length 20 to 40 bases separated by unique sequences of similar length [1-3], Fig. 1. These are conventionally known as Clustered Regularly Interspaced Short Palindromic Repeats (CRISPRs). The use of the term palindromic is somewhat misleading as the sequences are not palindromes, though some have short (4–8 base) terminal regions that are approximately or exactly reverse-complemented. CRISPRs have been implicated in DNA rearrangement [4], host cell defense, replication and regulation [5,6] and provide new tools for evolutionary study and strain typing [7]. Due to the high conservation and regular spacing of CRISPR repeats (Fig. 1), many of them can be found relatively easily using ad hoc software tools [2,6] or existing software tools such as Reputer [8], as used in [5], and PatScan [9], as used in [10]. However, the output of such tools is not necessarily ideal for this purpose, and significant post-processing and manual curation may be needed. We have therefore developed PILER-CR, a dedicated software tool for the identification and preliminary analysis of CRISPR repeats. We will present validation suggesting that PILER-CR has both high sensitivity and high specificity.

## Implementation

### Algorithm

PILER-CR is designed to identify the characteristic signature of CRISPR repeats. Once a repeat has been identified by PILER-CR, more sensitive search tools such as BLAST can be used to find fragmented or degraded copies of the repeat family. PILER-CR builds on techniques developed in the PILER family of algorithms for repeat analysis [12]. We will generically refer to the input sequence(s) as the *genome*. It may be useful to use several genomes as input as this will result in related repeats being clustered together (step 6 below). An *array *is a single CRISPR locus, i.e. set of CRISPR repeats and the intervening unique sequences known as *spacers*. The goal is to find a chain of local alignments that meet the criteria for being a CRISPR array, i.e. the repeats and spacers are within the expected ranges of length and sequence conservation. These ranges are parameters of the algorithm and can be changed by the user (Table 2).

**Table 2:** Major parameters of the PILER-CR algorithm

**Parameter**	**Description**	**Default**
minrepeat	Minimum length of a repeat. Smaller values might increase sensitivity, though repeats < 16 bases are not currently known.	16
maxrepeat	Maximum length of a repeat. Larger values might increase sensitivity, though repeats > 64 bases are not currently known. Larger values may also tend to give more false positives, such as RNAs.	64
minspacer	Minimum spacer length.	8
minarray	Minimum number of repeats in an array. A value < 3 will give large numbers of false positives. A value > 3 might increase specificity at the expense of missing shorter arrays.	3
maxspacer	Maximum spacer length.	64
minrepeatratio	Repeat length ratio (see caption for definition of ratio).	0.9
minspacerratio	Spacer length ratio (see caption for definition of ratio).	0.75
mincons	A run of minarray repeats with at least mincons similarity is required for an array to be reported. The value is specified as fractional identity 0.0 .. 1.0, with 1.0 meaning identical sequences. Increasing this value will tend to increase specificity by reducing the number of degraded or mutated repeats reported.	0.90

This table describes the most important parameters of PILER-CR algorithm. The Parameter column gives the name of the command-line option for the pilercr program. "Ratios" (*minrepeatratio *and *minspacerratio*) are defined as the ratio of the smallest to the longest, thus a value close to 1.0 requires lengths to be similar, 1.0 means identical lengths. Spacer lengths sometimes vary significantly, so the default ratio is smaller than for repeat lengths. Using a value of 1.0 for *minrepeatratio *or *mincons *is not recommended as this may lead to false negatives due to boundary mis-identification.

### Step 1: Local alignments

The first step is to find local alignments (hits) of the genome to itself; each hit is a candidate for being an alignment between two copies of a CRISPR repeat. As CRISPRs are separated by short distances, it suffices to search for alignments of two regions that are close to each other. Viewed as a self-similarity plot (dot-plot), this corresponds to searching for hits within a band around the main diagonal of the plot. PILER-CR uses a variant of Rasmussen *et al.*'s filter [13] to reduce the dynamic programming space. Searching within a band is accomplished by sliding a window along the genome. The filter utilizes a word index giving the sequence coordinates of each unique word. The index (occurrence table and lookup table, in Rasmussen *et al.*'s terminology) is updated in the obvious way as the window moves: words on the leading edge of the window are added to the index, words on the trailing edge are deleted. Regions of the dynamic programming matrix remaining after application of the filter are then explored for local alignments. Those local alignments that are too short or too long to plausibly align two CRISPRs are discarded. As an optimization, this is done during the dynamic programming step so that time is not wasted constructing long alignments.

### Step 2: Pile construction

**Figure 2:**
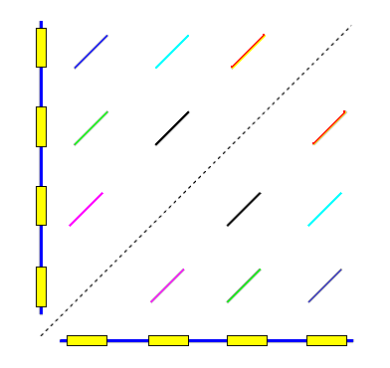
**Dot-plot of a CRISPR array against itself**. Self-similarity plot ("dot-plot") of a genome against itself in a CRISPR array region. The main diagonal is shown as a dashed line. As the two axes represent the same sequence, local alignments (diagonal lines) are symmetrical about the main diagonal.

**Figure 3:**
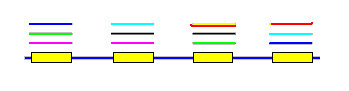
**Pile construction**. When local alignments are projected onto the genome, "piles" are produced. A pile is a contiguous sequence of bases, each one of which has a hit to at least one other region in the genome. Bases that are not in a pile are unique sequence. Each local alignment connects two piles. In this figure, each hit has a different color so, for example, the purple hit connects the first and second pile.

A *pile *is a contiguous set of bases, each one of which is covered by at least one local alignment. In other words, a pile is a repetitive region. By definition, piles are separated by unique regions, i.e. regions with no local alignments. As CRISPR repeats are separated by unique regions, a pile cannot contain more than one repeat. Piles are identified by sorting hit begin- and end-points by genome position, then traversing from beginning to end of the genome, keeping track of the coverage count at each point (add one when a begin point is encountered, subtract one when an end point is encountered). A pile begins where the coverage count rises above zero, and ends when it falls to zero again. Associated with each pile is the set of hits that cover it (Figs. 2 and 3).

### Step 3: Graph construction

Each hit connects two piles. Using hits as edges and piles as nodes, a graph is constructed and the connected components of the graph are identified. Each connected component is a candidate for containing one or more CRISPR arrays. This is not essential but significantly reduces the search space for the following step.

### Step 4: Draft array identification

**Figure 4:**
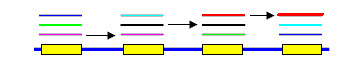
**A chain of hits meeting CRISPR criteria**. CRISPR arrays are identified by following chains of hits. Starting with a given pile, each hit that connects this pile to another pile later in the genome is a potential link in the chain. All possible chains are explored, abandoning the search each time the chain violates the criteria used for CRISPR array recognition (see Table 2). These criteria include maximum and minimum repeat length, maximum and minimum spacer length, and measures of the variance in repeat and spacer lengths. Shorter links are explored before longer links as regularly spaced arrays will be obtained by skipping every second, third... repeat. The figure shows the correct chain (arrows) for the example array from Fig. 3.

Within each connected component, each pile is tested as the possible first member of a putative CRISPR array by following each possible chain of local alignments, abandoning the search as soon as the array fails the test criteria (Fig. 4). Hits with the shortest spacers are considered first (because a set of hits that skips every *k*th repeat for a small integer *k *may also meet the criteria for being an array). When an array is positively identified, hits in the range of coordinates covered by the array are discarded and a search for additional arrays in any remaining hits continues. A multiple alignment of the pile sequences is constructed using fast options of the MUSCLE algorithm [14,15], and a consensus sequence is generated.

### Step 5: Array refinement

Given output from step 4, several heuristics are applied in an attempt to improve the inferred arrays.

5a. Partial or degraded copies of the repeat at the beginning or end of the array are deleted. These are often found in practice, and deleting them may expose conserved columns in flanking regions that correctly belong in the repeat. A partial or degraded repeat is identified by having three or more gaps when aligned to the current consensus sequence for the array, or by having more than twice as many gaps as the average for the array. This is repeated until there are no such repeats.

5b. A 100% conserved column immediately preceding or following the current boundary is moved into the repeat. This is repeated until there are no such columns.

5c. A column at the repeat boundary that is < 90% conserved is deleted from the repeat, i.e. moved into the flanking region. This is repeated until there are no such columns.

### Step 6: Adjacent array merge

Adjacent arrays with similar consensus sequences and similar spacer lengths are merged. Here, "merging" means that the two arrays are reported as a single array. The reason for this step is that it is quite common for there to be one or a small number of missing or degraded repeats embedded in an array. For two arrays to be merged, the following criteria must all be met. The ratios (a) of the repeat length and (b) spacer lengths for the two arrays must be ≥ 0.95. The two consensus sequences must be globally alignable, where global alignability is defined as having an optimal local alignment (Smith-Waterman) that covers ≥ 95% of the bases in the first array. Finally, the distance between the end of the first array and the beginning of the second array must be approximately *k*(*R *+ *S*), where *k *is 1, 2, 3 or 4, *R *is the average repeat length of the first array, *S *is the average spacer length, and "approximately" means that the ratio must be ≥ 0.95.

### Step 7: Clustering and alignment

It is often found that repeats in different arrays have recognizably similar sequences. The program therefore clusters consensus sequences into similar groups, meaning groups of sequences having estimated mutual identities of ≥ 50%. For speed, the identity of two (unaligned) sequences is estimated by counting the number of identical words of length 4. Multiple alignments are created for each group, again using fast options of MUSCLE.

### Step 8: Report generation

Finally, a report is generated; for an example see Additional file 2. A detailed section specifies each repeat in an array, indicating any differences from the consensus sequence. Summary sections group arrays by estimated sequence similarity (see Step 7 above) and by position in the genome.

### Complexity

The time and space complexities of the algorithm are strictly O(*L*^3^) where *L *is the genome length. Steps 1 though 3 are O(*L*^2^) under reasonable assumptions; the cubic term arises in Step 4. In practice, time and space requirements scale approximately as O(*L*).

### Estimating sensitivity

The most common error made by PILER-CR is to mis-identify the endpoints of a CRISPR repeat by one or more positions (rarely more than one). However, in most such cases the array is essentially correctly identified, and the correct endpoints are immediately evident to the user from the report. When comparing two repeat sequences, we therefore define three categories of match: an exact match, a *close *match and no match (meaning not exact or close). An exact match requires the two sequences to be identical. A close match is defined by the following procedure. A local alignment of the two sequences is made using the Smith-Waterman algorithm, and the number of matching bases *m *in this alignment is computed. If the longest of the two full sequences has length *L*, the identity of the two sequences is defined as *s *= *m*/*L*. (Note the difference between this identity and the conventionally defined identity of the local alignment, which in general will be shorter and thus have higher identity). If *s *exceeds a pre-defined threshold, which we choose to be 0.9, then we consider the match to be close.

### Estimating specificity

While false positives cannot be reliably determined in all cases, due to the lack of known complete reference data, some can be identified by visual inspection or by searches of databases of other types of repeat. Some RNA genes occur in arrays that are remarkably similar to CRISPR repeats, with the gene sequence and spacer lengths 100% conserved; these are occasionally reported by PILER-CR as CRISPRs.

## Results

Here we report the results of running PILER-CR on 346 prokaryotic genomes (Additional file 1, Tables 1 and 2), and compare the reported CRISPR repeats with two previous surveys due to Jansen *et al*. [10] and Godde and Bickerton [11] (Table 1, also Additional file Tables 1 and 2). For brevity, we shall refer to these studies as J and G respectively. While J appears to be an attempt to identify all CRISPR repeats in the studied species, G is limited to CRISPRs that are associated with *cas *genes. We can thus use a comparison with J as a guide to both the sensitivity and specificity of PILER-CR, while G can be used as a guide to sensitivity only (a repeat in G should be found by PILER-CR, but the converse is not the case). We also reviewed each array manually and attempted to assess whether the array itself appeared to be a CRISPR, and if so whether the endpoints were correctly identified. While we believe this validation strategy gives a good qualitative overview of the accuracy of PILER-CR, it should not be considered rigorous. There is no independent experimental technique for identifying "true" CRISPR arrays, so we are comparing different computational methods against each other, each one of which has *a priori *unknown accuracy. Also, as accession numbers are not given in J or G, the input sequences used in the different studies may vary due to differences in the strains being sequenced, further data being added to partially sequenced genomes, and differences in draft versus finished genomes. It should also be noted that there is a subjective element in evaluating arrays, especially when they are short (three or four elements long). Finally, the entire J and G sets were used to assist in algorithm parameter tuning. A more rigorous strategy might divide J and G into test and training sets, though we see limited value in this approach given the lack of independent experimental evidence.

**Table 1:** Comparison of PILER-CR results with the reference sets due to Jensen *et al. *and Godde and Bickerton

**Reference set**	**Exact**	**Close**	**No Match**
Jensen *et al*.	34	19	6
Godde and Bickerton	79	19	11

This table shows a comparison between repeats found by PILER-CR compared with those found by Jensen *et al. *and by Godde and Bickerton. See Estimating Sensitivity for definitions of the categories Exact, Close and No Match. This shows that PILER-CR finds exact or close matches with most sequences in the reference sets. Of the No Match cases, most appear to be due to differences in the genome sequences used in this study versus the previous study, most likely due to the use of different strains.

### Sensitivity

Additional file 1, Table 1 summarizes the agreement between CRISPR sequences reported by PILER-CR and those in the G and J sets (see Estimating Sensitivity in Methods). In some cases, we found no instances of the sequences reported by G or J in the genome, which is presumably explained by differences in the sequences used here versus the other studies. In other cases, all in the J set, PILER-CR found longer sequences that include the reference repeat as a subsequence, suggesting that Jensen *et al. *may have failed to identify the full repeat sequence. In a few cases, PILER-CR concatenates two arrays with similar repeat sequences, causing the consensus sequence to be truncated. There were no unambiguous false negatives. Thus, the sensitivity of PILER-CR with default parameters may approach 100%.

### Specificity

Again, Additional 1, file Table 1 is used (see Estimating Specificity in Methods). If longer lengths are allowed for CRISPR repeats, this may result in false positives due to RNA genes; the one example found in our experimental data with default algorithm settings is the longest repeat in *Chromohalobacter salexigens*. In Additional file 1, Table 1 we have marked clear false positives as FP and questionable repeats as Q. We found 8 questionable and 11 false positives in a total of 319 repeats. We assume that repeats classified as close or truncated (see methods), or are novel versus the reference data, are true positives as there is no reason to suppose otherwise. If we further assume that all the questionable repeats are errors, we get an estimated specificity of 94%. This estimate is necessarily very approximate, and may be quite optimistic.

## Conclusion

We have presented PILER-CR, a new software program for the identification of CRISPR repeats, and have shown that the algorithm probably has both good sensitivity and good specificity. While other repeat-finding methods can be applied to finding CRISPRs, sometimes requiring post-processing of their output, PILER-CR provides an integrated package generating a comprehensive and readable report. The program executes rapidly, completing the analysis of 346 prokaryotic genomes in about 15 minutes on a 2 GHz desktop computer. The output is designed to facilitate manual analysis of the reported CRISPR arrays. In addition to a multiple alignment of the repeats in each array, flanking regions and spacers are shown, making it visually obvious when PILER-CR mis-identifies the repeat boundary by one or a few positions. Repeats are clustered by similarity, exposing relationships between different arrays and, where applicable, providing confirmatory evidence of marginal arrays (i.e., arrays with only 3 or 4 repeats that may not be perfectly conserved).

## Availability and requirements

PILER-CR is donated to the public domain. Source code is written in C++ and is designed to be easily ported across platforms. It has been compiled and tested using Microsoft Visual C++ on Windows and gcc on i86 Linux. Source code and a Linux binary are freely available as Additional File 3, which contains the version used in this study, and at , where any updates and bug fixes will be posted.

## Authors' contributions

RCE is solely responsible for the manuscript and the work described herein.

## Supplementary Material

Additional File 2 Sample output generated by PILER-CR. The report has three sections: Detailed, Summary by Similarity and Summary by Position. The detailed section shows each repeat in each putative CRISPR array. The summary sections give one line for each array. Columns in the detailed section are: *Pos*, sequence position; *Repeat*, length of the repeat; *%id*, identity with the consensus; *Spacer*, length of spacer to the right of this repeat; *Left flank*, 10 bases to the left of this repeat, *Repeat*, sequence of this repeat (dots indicate positions where this repeat agrees with the consensus sequence below); *Spacer*, sequence of spacer to the right of this repeat, or 10 bases if this is the last repeat. The left flank sequence duplicates the end of the spacer for the preceding repeat; it is provided to facilitate visual identification of cases where the algorithm does not correctly identify repeat endpoints. At the end of each array there is a sub-heading that gives the average repeat length, average spacer length and consensus sequence. Columns in the summary sections are: *Array*, number 1, 2 ... referring back to the detailed report; *Sequence*, FASTA label of the sequence; *From*, start position of array; *To *end position of array; *# copies*, number of repeats in the array, *Repeat*, average repeat length; *Spacer*, average spacer length; +, +/-, indicating orientation relative to the first array in the group, *Distance*, distance from previous array; *Consensus*, consensus sequence. In the Summary by Similarity section, arrays are grouped by similarity of their consensus sequences. If consensus sequences are sufficiently similar, they are aligned to each other to indicate probable relationships between arrays. In this example, Arrays 1–5 are very similar and are thus aligned; Array 6 appears to be unrelated and stands alone. In the Summary by Position section, arrays are sorted by position within the input sequence file. The Distance column facilitates identification of cases where a single array has been reported as two adjacent arrays. In such a case, (a) the consensus sequences will be similar or identical, and (b) the distance will be approximately a small multiple of the repeat length + spacer length. In the above example, we see how the flanking sequences provide immediate visual feedback. Array 4 has only three repeats, and the last column in the repeat alignment is (ACA), i.e. is not conserved. This column should probably be deleted from the repeat and moved to the spacer. Array 1 has a similar issue, but here it is not so clear that the last column should be deleted. The first three repeats in the array are perfectly conserved, and it is common to find degraded copies of the repeat at the beginning and end of an array. This also illustrates the difficulty of developing heuristics that are able to match human performance in making judgments in more difficult cases. Click here for file

Additional File 1 Additional file Tables 1 and 2 Click here for file

Additional File 3 Tar with gzip compression. Source code and i86 Linux binary. Click here for file
