# Supplementary material for: PILER-CR: Fast and accurate identification of CRISPR repeats
Source: BMC Bioinformatics. 2007 Jan 20;8:18. doi: 10.1186/1471-2105-8-18 (PMC1790904; doi:10.1186/1471-2105-8-18)
Supplement: Additional File 1 — Additional file Tables 1 and 2 [file 1471-2105-8-18-S1.doc]

Supplementary material for Edgar, R.C., *PILER-CR: Fast and accurate identification of*

*CRISPR repeats*.

### Supplementary Table 1

| **Species** | **Repeat** | **Notes** |
| --- | --- | --- |
| Acinetobacter calcoaceticus | TTTCTAAGCTGCCTGTGCGGCAGTTAAG |  |
|  | CTTCACTACCGCACAGGTAGCTTAGAAA |  |
|  | GTTCGTCATCGCATAGATGATTTAGAAA |  |
| Acinetobacter sp ADP1 | TTTCTAAGCTGCCTGTGCGGCAGTTAAG |  |
|  | CTTCACTACCGCACAGGTAGCTTAGAAA |  |
|  | GTTCGTCATCGCATAGATGATTTAGAAA |  |
| Aeropyrum pernix | GAATCTTCGAGATAGAATTGCAAG | G J |
|  | GCATATCCCTAAAGGGAATAGAAAG | J |
|  | CTTGCAATTCTATCTCGAAGATTC | j |
| Anabaena variabilis ATCC 29413 | GTTTTAATTAACAAAAATCCCTATCAGGGATTGAAAC | j |
|  | AGTTTCAACACCCCTCCCGAAGTGGGGCGGGTTGAAAG | Q |
|  | GTTTCAATCCCTGATAGGGATTTTAGAGGGTTTTAAC | J+ |
|  | aagGTGACAATAGCCCTTCCCGTGTTGAGCGGGTTGAAAGg | J+ |
|  | GTGCTTTAACATTAGATGTCGTTAGGCGTTGAGCAGG | J+ |
|  | ATTGCAATTAACTAAAATCCCTATCAGGGATTGAAAC | G J+ |
| Aquifex aeolicus (J-) | GTTCCTAATGTACCGTGTGGAGTTGAAACc |  |
|  | GTTTCAACTCCACACGGTACATTAGGAAC | G J |
|  | GTTTCTAATGTACCGTAGAGGAGTTGAAACc |  |
|  | GTTTCAACTCCACTACGGTACATTAGGAAC | J |
|  | CTTTCTATCCCATATATGGGAACTAAAAC | J+ |
| Archaeoglobus fulgidus | GTTGAAATCAGACCAAAATGGGATTGAAAG | G |
|  | CTTTCAATCCCATTTTGGTCTGATTTCAAC |  |
|  | GTAAGAAAGGGAGGCTCCTGAAAATGGAGATTGAAAG | G J+ |
| Azoarcus sp EbN1 | GTTTCAATCCACGCCCCCCGTCACCGAGGGGCGATGC |  |
|  | GTGTTCCCCGCGCATCGCGGGGGTTGAAG |  |
| Azotobacter vinelandii | cCGGTTCATCCCCGCACC |  |
|  | ACGGTTCATCCCCGCACCCgcggggaacgcgctt |  |
|  | GTTTCAATCCACACGCCCGCATGGGGCGTGAC |  |
| Bacillus cereus ATCC14579 | TGCTGCTGCCTTCGCCTTCGCTTTCGCTTTTTCTTCTTCCGTTACTTCTTCGGT |  |
| Bacillus clausii KSM-K16 | ATTTCAATCCACGCACTCACAAAGAGTGCGAC | G |
|  | ATTTCAATCCACGCACTCAC |  |
| Bacillus halodurans | GTCGCACTCTACATGAGTGCGTGGATTGAAAT | G |
| Bacteroides fragilis NCTC 9434 | ATTTCAATTCCATAAGGTACAATTAATAC | G |
|  | GTTGTGATTTGCTTTCAAATTAGTATCTTTGAACCATTGGAAACAGC | G |
| Bacteroides fragilis YCH46 | ATTTCAATTCCATAAGGTACAATTAATAC |  |
| Burkholderia 383 | AGGTGAGATTTTTCGGGA | FP |
| Campylobacter jejuni | tGTTTTAGTCCCTTTTTAAATTTCTTTATGGTAAAAT | g j |
| Campylobacter jejuni RM1221 | TGTTTTAGTCCCTTTTTAAATTTCTTTATGGTAAAAT | g |
| Carboxydothermus hydrogenoformans Z-2901 | {G,TTT}CAATCCCAGAATGGTTCGATTAAAAC |  |
|  | GTTTCAATCCCAGATTGGTTCGATTAAAAC | j |
| Chlorobium chlorochromatii CaD3 | GTTTCAATCCACGCGCCCGCGAAGGGCGCGAC |  |
|  | ATTTCAATTCCATATTGGTGCAATTAGAAG |  |
| Chlorobium tepidum TLS | GTTTCAATCCACGCGCCCGCGCGGGGCGCGAC | G J |
|  | GTCTTCCCCACGCCCGTGGGGGTGTTTC | G J+ |
| Chloroflexus aurantiacus | CTTCAAAAAACCAAAATCCCCGCGAGGGGATTGAAAA |  |
|  | CAGCAGAGCATTGCCCCGCAATGAAGGGGTTTGAAAC |  |
|  | CTTTCAACAATTTCGGCTCACGGTAGAGCACTGAAAC |  |
|  | {G,T}TTTCAATCCCCTCGCGGGGATTTTGGTTTTTTGAAG |  |
|  | GTTTCAGTGCTCTACCGTGAGCCGAAATTGTTGAAAG |  |
| Chromobacterium violaceum | GTCGCGCCCAACGCGGGCGCGTGGATTGAAAC |  |
|  | GTGTTCCCCACGTACGTGGGGATGAACCG | G |
|  | TTTCTAAGCTGCCTATCCGGCAGTGAAC | g |
| Chromohalobacter salexigens DSM 3043 | CCGTTCCCCGCAGGCGCGGGGATCAACCG |  |
|  | tCCGTTCCCCGCAGGCGCGGGGATCAACCGt |  |
|  | CGTTCCCCGATAGCTCAGTTGGTAGAGCAAATGACTGTTAATCATTGGGTCGCAGG | FP |
| Clostridium difficile QCD-32g58 (J-) | GTTTTATATTAACTAAGTGGTATGTAAAG |  |
|  | CTTTACATTCCATATAGTTAATATAAAAC | J+ |
|  | ATTTATAACTAACTTAGTGTAATTTAAAC | J+ |
| Clostridium tetani E88 | GTATTAGTAGCACCATATTGGAATGTAAAT | G |
|  | ATTTAAATACAACTCTTGTTATTGTTCAAC | G |
|  | gaATTTAAATACATCCTATGTTAAGGTTCAAC |  |
| Clostridium thermocellum | GTTTCAATCCTTGTTTTACTGGAAGTACCTCTTCAAC |  |
|  | TTTCAATTCCTCATAGGTACGATACAAAC |  |
|  | GTTTCAATTCCTCATAGGTACGATACAAAC |  |
|  | GTTTTTATCGTACCTATGAGGAATTGAAAC |  |
| Corynebacterium diphtheriae | GAAGTCTATCAGGGTTTTTGAGAACTGAACCCCAGT | g j |
|  | GTCTTCTCCGCACACGCGGAGGTATTTC | G |
| Corynebacterium efficiens YS-314 | CCCTCAATGAAAGGCCGTCGAACTAACGACGGCAGG |  |
| Corynebacterium jeikeium K411 | GGCTCATCCCCGCTGGCGCGGGGAGCAC |  |
| Cyanobacteria bacterium Yellowstone A-Prime | CGGTTCACCCCCACGGGTGTGGGGACAAC |  |
|  | GTTTCCGTCCCCTTGCGGGGAAAAGGTAGGGATCAAC |  |
|  | GTTCCCCCTTCGGGGGGATCCCTAGAAATTGGAAAC |  |
|  | GTTTCCAATTTCTAGGGATCCCCCCGAAGGGGGAAC |  |
|  | TGTTTCCAATTTCTAGGGATCCCCCCGAAGGGGGAACCCT |  |
| Cyanobacteria bacterium Yellowstone B-Prime | GTTCCCCCTTCGGGGGGATCCCTAGAAATTGGAAAC |  |
|  | GTTTCCAATTTCTAGGGATCCCCCCGAAGGGGGAAC |  |
|  | GTTTCCGTCCCCTTGCGGGGAAAAGGTAGGGATCAAC |  |
| Dehalococcoides CBDB1 | CGGTTCACCCCCACATGCGTGGGGAATAC |  |
| Desulfitobacterium hafniense Y51 | GTTTCAATCCCTTATAGGTAAGCTAACAAC | G |
|  |  |  |
| Desulfotalea psychrophila LSv54 | CTTCAATGTAGTCACCCCTTTCGAGGTGATTGATAC | g |
| Desulfovibrio desulfuricans G20 | GGTTCATCCCCGCGGGTGCGGGGAACAC | g |
| Desulfovibrio vulgaris Hildenborough | GTCGCCCCCCACGCGGGGGCGTGGATTGAAAC |  |
| Erwinia carotovora atroseptica SCRI1043 | TTTCTAAGCTGCCTGTACGGCAGTGAAC | G |
|  | GTTCACTGCCGTACAGGCAGCTTAGAAA |  |
| Escherichia coli K12 | CGGTTTATCCCCGCTGGCGCGGGGAACTC | g J |
|  | GGTTTATCCCCGCTGGCGCGGGGAACAC | j |
| Escherichia coli O157H7 | CGGTTTATCCCCGCTGGCGCGGGGAACACa | g j |
| Escherichia coli O157H7 EDL933 | CGGTTTATCCCCGCTGGCGCGGGGAACACa | g j |
| Escherichia coli UTI89 | GTTCACTGCCGTACAGGCAGCTTAGAAA |  |
| Escherichia coli W3110 | CGGTTTATCCCCGCTGGCGCGGGGAACTC |  |
|  | GGTTTATCCCCGCTGGCGCGGGGAACAC |  |
| Exiguobacterium 255-15 | ATTTCAATCCACGCACTCACGAGGAGTGCGAC |  |
| Frankia CcI3 | GTCGTCCCCGCACGCGCGGGGATCTTCC |  |
|  | GTTGTGATCCTCGCCGAGGGCGATCCCTCGGCGCTGC |  |
|  | GCAGCGCCGGGCGTCCGCGCCCGGCGAGGTTCCCAAC |  |
| Fusobacterium nucleatum | ATTTAAATTCTAATATAGAAATACATAAAT | G |
| Geobacillus kaustophilus HTA426 | GTTTTTATCGTACCTATGAGGGATTGAAAC |  |
|  | GTTTCAATCCCTCATAGGTACGATAAAAAC |  |
| Geobacter metallireducens GS-15 | GTAGCGCCCGCCTACATAGGCGGGCGAGGATTGAAAC | G |
| Geobacter sulfurreducens | GTATTCCGGGGCCATGATGCCCCGGCCTCATTGAAGC | G J |
|  | GTGTTCCCCGCATGCGCGGGGATGAACCG |  |
| Hahella chejuensis KCTC 2396 | GTCGCCTCCCACGCGGAGGCGTGGATTGAAAC |  |
| Haloarcula marismortui ATCC 43049 | GCTTCAACCCCACAAGGGTCCGTCTGAAAC |  |
|  | GTTACAGACGGACCCTCGTGGGGTTGAAGCtc |  |
|  | ACACCACCCTGCAAGTGTT | FP |
| Lactobacillus acidophilus NCFM | GGATCACCTCCACATACGTGGAGAAAA |  |
| Lactobacillus salivarius UCC118 | GTTTCAGAAGTATGTTAAATCAATAAGGTTAAGACC{T,C} |  |
| Legionella pneumophila Lens (JZ) | GTTCACTGCCGCACAGGCAGCTTAGAAA | G |
|  | TTTCTAAGCTGCCTGTACGGCAGTGAAC |  |
| Leptospira interrogans serovar Copenhageni | tccGTGCTCAACGCCTAACGGCATCAAAGTTATATTCAG |  |
| Leptospira interrogans serovar Lai | TCTGAATATAACTTTGATGCCGTTAGGCGTTGAGCACac |  |
|  | CTGAATATAACTTTGATGCCGTTAGGCGTTGAGCAC | G |
| Listeria innocua | GTTTTGTTAGCATTCAAAATAACATAGCTCTAAAAC | G |
| Listeria monocytogenes | GTTTTAGTTACTTATTGTGAAATGTAAAT |  |
| Magnetococcus sp MC-1 | GTTTCAATCCACGCCCCCGTGGGAGGGGGCGAC |  |
| Mannheimia succiniciproducens MBEL55E | GTTTCAATTCACGCTCTCGTGTGAGAGCGGAG | G |
|  | GTTTCAATCCCTTTAAGACAGGGCAAGGTCTTTCGAC | g |
| Methanobacterium thermoautotrophicum | ATTTCAATCCCATTTTGGTCTGATTTTAAC | J |
|  | TACACTTGAAATGAATGTCTCcc | Q |
|  | GTTAAAATCAGACCAAAATGGGATTGAAAT |  |
| Methanococcoides burtonii DSM 6242 | GAGTTCCCCATGCATGTGGGGATAAACCG | G |
|  | GTTAAAATCAGACCTTAGAGGGATTGAAAC | G |
| Methanococcus jannaschii | ATTAAAATCAGACCGTTTCGGAATGGAAA | G j |
|  | aTTTCCATCCTCCAAGAGGTCTTATTTTAAT | Q |
|  | GTTAAAATCAGACCTCTTGGAGGATGGAAA | G |
|  | TTTCCATCCTCCAAGAGGTCTGATTTTAAc |  |
| Methanopyrus kandleri | GTTTCATTACCCGTATTATTACGGGTTAATTGCGAG |  |
|  | CTCGCAATTAACCCGTAATAATATGGGTAATGAAAC | g |
| Methanosarcina acetivorans | ATTCGAGAGCAAGATCCACTAAAACAAGGATTGAAAC | G |
|  | GTTTCAATCCCTCTAAGGTCTGATTTTAAC |  |
| Methanosarcina barkeri fusaro | GTTAAAATCAGGCCTTAGAGGGATTGAAAC |  |
|  | TCCACTAAAATAAGGATTGAAAC | FP |
|  | ATTCGTGAGCAAGATCCACTAAAACAAGGATTGAAAC |  |
|  | GCTTCAATTCTGCCACAACCTTTCGGTTATGGAAAC |  |
| Methanosarcina mazei | GTTTCAATCCTTGTTTTAATGGATCTTGCTCGCGAAT | G |
| Methanosphaera stadtmanae | GTTTAAAATAGACTTAATAGTATGAAAAC |  |
|  | AATAGAATAAGATCATAATGAAATTGAAAT |  |
|  | AATGGAGGAGCAATATATAAT | Q |
| Methanospirillum hungatei JF-1 | GTTGCCATACCCTTCTATTTTCGGGTCACTTGCAAC |  |
|  | CGGTTCATCCCCATACACACGGGGAACTC |  |
|  | GTTTCAATCCCTATCGGGTTTTCTTTTCCATTGTGAC |  |
| Methylobacillus flagellatus KT | GTGTTCCCCGCACATGCGGGGATGAACCGgc | G |
|  | GTCGCACCCTGCGCGGGTGCGTGAATTGAAAC |  |
| Methylococcus capsulatus Bath | GTTTCAATCCACTCCCGGCTATTTAGCCGGGAGATAC | G J |
|  | GGTCTATCCCCGCGTGTGCGGGGGAGCC | G |
| Moorella thermoacetica ATCC 39073 | GTTTCAACCCTCGCCCGGCATGGAAGCCGGGCGCGAC | G |
|  | GTTCAAATTCCTCTATGGTCGATGGTCAC | G |
| Mycobacterium bovis | GTTTCCGTCCCCTCTCGGGGTTTTGGGTCTGACGAC | J |
| Mycobacterium tuberculosis CDC1551 | GTTTCCGTCCCCTCTCGGGGTTTTGGGTCTGACGAC | G J |
| Mycobacterium tuberculosis H37Rv | GTTTCCGTCCCCTCTCGGGGTTTTGGGTCTGACGAC | G |
| Mycoplasma capricolum ATCC 27343 | TTCAACCAACCCATTGGTAACTGAGACACCTCAAATGT | Q |
| Mycoplasma gallisepticum | GTTTTAGCACTGTACAATACTTGTGTAAGCAATAAC | G |
| Mycoplasma mobile 163K | GTTTAAGAATACATAAGAATGATACTACACCAAAAC | G |
| Mycoplasma synoviae 53 | GTTTTGGGGTTGTACAATTATTTTGTTAAGTAAAAC |  |
| Nanoarchaeum equitans | CTTTCAATATTTCTAATATATTAGAAAC | G |
| Natronomonas pharaonis | GTTTCAGACGAACCCTTGTGGGGTTGAAGC |  |
|  | GTCGAGACGGACTGAAAACCCAGAACGGGATTGAAAC |  |
|  | CACCCCTCTATCGATGTGTA |  |
| Neisseria meningitidis Z2491 | GTTGTAGCTCCCTTTCTCATTTCGCAGTGCTACAAT | G J |
| Nitrobacter winogradskyi Nb-255 | GTTTCGACCCACGCCCCCGCGAAGGGGGCGAC |  |
| Nitrosococcus oceani ATCC 19707 | GTTCACCGCCGCACAGGCGGTTTAGAAA |  |
| Nitrosomonas europaea | GTCTCAATCCCTTTGAAATCAGGGCATCGGTGTTTC | G |
|  | GTAGCGCCCGGTCACCAGACCGGGCGAGGATTGAAAC | G |
| Nitrosospira multiformis ATCC 25196 | CAAAGTCCGAGGTTTGGCTCCCAA | FP |
| Nocardia farcinica IFM10152 | CTCATCCCCGCATGCGCGGGGAGCAC |  |
|  | GGCTCATCCCCGCGTGCGCGGGGAGCAC |  |
|  | GGGCTCATCCCCGCACGCGCGGGGAGCAC | g |
|  | GCTCCCCGCGCACGCGGGGATGAGCC |  |
| Nostoc punctiforme | GTTGCAAAACACCTCATCCCTGATAGGGATTCAAAC |  |
|  | ATTGCAATTTATCAAAATCCCTATTAGGGATTGAAAC |  |
|  | GTTTCAATCCCTAATAGGGATTTTGAGAAATTGCAAT |  |
|  | CTTTCAACCCACCCAGTACCTGGAGGGTTGTTGCCAC |  |
| Nostoc sp | GTTACTTACCATCACTTCCCCGCAAGGGGATGGAAAC |  |
|  | GTTTCCATCCCCGTGAGGGGTAAAGGAATTAAAAC |  |
|  | GTTTTAATTCCTTTACCCCTCACGGGGATGGAAAC |  |
|  | GTTTCTATTAACACAAATCCCTATCAGGGATTGAAAC |  |
|  | GTTTCCATCCCCGTGAGGGGTAAGAGATTAAAAAC |  |
|  | TTCAATCCCTGATAGGGATTTTTGTTAGTTAAAAC |  |
|  | GTTTCAATCCCTGATAGGGATTTTTGTTAGTTAAAAC |  |
|  | GTTGCAACACCATATAATCCCTATTAGGGATTGAAAC |  |
|  | CTTTCAACCCTCCCATTACTGGAAGGAGGGTTGCAACG |  |
|  | GTTTCCATCCCCTTTCGGGGTGATGTGATCGGAAAG |  |
|  | GTTAAAACCCTCTAAAATCCCTATCAGGGATTGAAAC |  |
| Parachlamydia sp UWE25 | AATCTCACTGATGCTGGATTAGCGCATTTGACACCCTTAGTCGCTTT | FP |
| Pasteurella multocida | GTTCACCATCGTGTAGATGGCTTAGAAA | J |
|  | GTTGTAGTTCCCTCTCTCATTTCGCAGTGCTACAAT | g |
|  | GTTAACTGCCGTATAGGCAGCTTAGAAA |  |
| Pelodictyon luteolum DSM 273 | GTCGCGCCCCACGCGGGCGCGTGGATTGAAAC |  |
| Photobacterium profundum SS9 | TTTCTAAGCTGCCTGTGCGGCAGTGAAC | G |
|  | CGGTTCAGCCCCGTGAGTACGGGGAACAC |  |
| Photorhabdus luminescens | GTGCACTGCCGTACAGGCAGCTTAGAAA | G |
| Picrophilus torridus DSM 9790 | ACTTTCAATCCTATTTAGGTTATTATTTAAC |  |
|  | CTTCCATACTATCTAGTAATTCTTAAAC | G |
|  | CTTTCAATCCTATTTAGGTTATTATTTAAC |  |
| Porphyromonas gingivalis W83 | GTTGGATCTACCCTCTATTCGAAGGGTACACACAAC | G J |
|  | GTTGTCTCCACCCTTCTAACTAAGGGTATTCCCAAC |  |
|  | GTCTTAATAGCCTTACGGACTGTGTATGTATAGTGAG |  |
|  | GTTTTAATTCCTGTATGGTGCAATTGAAAT |  |
| Pseudomonas syringae pv B728a | CTGAGTTCGGAATCCGAACTC | FP |
| Pyrobaculum aerophilum | GTTTCAACTATCTTTTGATTTCTGG | G |
|  | CCAGAAATCAAAAGATAGTTGAAAC |  |
|  | GAATCTCAAAAAGAGGATTGAAAG | G |
|  | GTTTCAATTCTTTTGTAGATTCTTC |  |
| Pyrococcus abyssi | CTTTCAATTCTATTTTAGTCTTATTGGAAC |  |
|  | CTTTCCACACTACTAAGTTCTACGGAAAC |  |
|  | GTTCCAATAAGACTAAAATAGAATTGAAAG | j |
| Pyrococcus furiosus | GTTCCAATAAGACTAAAATAGAATTGAAAG | G J |
|  | CTTTCAATTCTATTTTGGTCTTATTGTAAC | G |
|  | CTTCAATTCTTTTGTAGTCTTATTGGAAC | j |
| Pyrococcus horikoshii | CTTTCCACACTATTTAGTTCTACGGAAAC | J |
|  | GTTTCCGTAGAACTTAGTAGTGTGGAAAG | g |
|  | GTTCCAATAAGACTATAAGAGAATTGAAAG |  |
|  | TTTCCACACTATTTAGTTCTACGGAAAC |  |
|  | CTTTCAATTCTATTTTAGTCTTATTGGAAC |  |
| Rhodoferax ferrireducens DSM 15236 | GGATCGCCCGGCATTCATGTCGGGCGCGGATTGAAAC |  |
|  | CTTCAATCCCCGCGCAACGCGGGGCGC |  |
| Rhodopseudomonas palustris BisB18 | GCCGTGGCTTCCCTACCGATTTCCCCGTGGTAGGCT |  |
| Rhodopseudomonas palustris BisB5 | AGCCTACCACGGGGAAATCGGTAGGGAAGCCACGGC |  |
| Rhodospirillum rubrum ATCC 11170 | CGGTTCATCCCCGCGCATGCGGGGAACAGC | G |
|  | GTTTCAATGAGGCCGAGGCATTACTGCCTCGGGAGAC | G |
|  | GGTTCCCCCGCACTCGTGGGGATAGACC |  |
|  | ACTCTACCATGGCGGTGTGGGACGGGGCCATGGAAC |  |
|  | GTCGCCCCCTTCACGGGGGCGTGGATCGAAAC |  |
|  | GTTTCAGTCCCCTCGATGGCGGGGCATAGGGTGCGAG | g |
|  | GTTTCAATCCACGCCCCCGTGAGGGAGCGAC | G |
|  | CTCGCAGCCTATGCCCCGCCACCGAGGGGACTGAAAC |  |
|  | GTCTCCCGAGGCAGTAATGCCTCGGCCTCATTGAAAC | G |
| Rickettsia felis URRWXCal2 | TAATCTTGATAAAGCAATAGAGAAAGGTGATTTAGCT | FP |
| Salmonella enterica Choleraesuis | GTTTATCCCCGCTGGCGCGGGGAACA |  |
|  | CGGTTTATCCCCGCTGGCGCGGGGAACAC |  |
| Salmonella enterica Paratypi ATCC 9150 | CGGTTTATCCCCGCTGGCGCGGGGAACAC | g |
| Salmonella typhi | CGGTTTATCCCCGCTGGCGCGGGGAACAC | G J |
| Salmonella typhimurium LT2 (J-) | GGTTTATCCCCGCTGGCGCGGGGAACAC |  |
|  | CGGTTTATCCCCGCTGGCGCGGGGAACAC |  |
| Salmonella typhi Ty2 | CGGTTTATCCCCGCTGGCGCGGGGAACAC |  |
| Staphylococcus epidermidis RP62A | GTTCTCGTCCCCTTTTCTTCGGGGTGGGTATCGATCc | g |
| Streptococcus agalactiae 2603 | GTTTTAGAGCTGTGCTGTTTCGAATGGTTCCAAAAC | G |
| Streptococcus agalactiae A909 | GTTTTAGAGCTGTGCTGTTTCGAATGGTTCCAAAAC |  |
| Streptococcus agalactiae NEM316 | GTTTTAGAGCTGTGCTGTTTCGAATGGTTCCAAAAC | G |
| Streptococcus mutans | GTTTTGGAACCATTCGAAACAACACAGCTCTAAAAC | G J |
| Streptococcus pyogenes M1 GAS | GTTTTAGAGCTATGCTGTTTTGAATGGTCCCAAAAC | G J |
|  | tATTTCAATCCACTCACCCATGAAGGGTGAGACt | j |
| Streptococcus pyogenes MGAS5005 | GTTTTAGAGCTATGCTGTTTTGAATGGTCCCAAAAC |  |
|  | ATTTCAATCCACTCACCCATGAAGGGTGAGAC |  |
| Streptococcus pyogenes MGAS6180 | GTTTTAGAGCTATGCTGTTTTGAATGGTCCCAAAAC |  |
| Streptococcus suis | GTTTTACTGTTACTTAAATCTTGAGAGTACAAAAAC |  |
| Streptococcus thermophilus CNRZ1066 | GTTTTTGTACTCTCAAGATTTAAGTAACTGTACAAC | G |
| Streptococcus thermophilus LMG 18311 | GTTTTTGTACTCTCAAGATTTAAGTAACTGTACAAC | G |
|  | GATATAAACCTAATTACCTCGAGAGGGGACGGAAAC |  |
| Streptomyces avermitilis | CGACCCACCTCCGCTCGCGCGGAGAG{CAC,AAC} |  |
|  | CGACCCACCTCCGCTCGCGCGGAGAGAAC |  |
|  | GTGCTCTCCGCGCGAGCGGAGGTGAACCG | G |
| Sulfolobus acidocaldarius DSM 639 | GTTTTAGTTTCTTGTCGTTATTAC |  |
|  | GTAATAACGACAAGAAACTAAAAC |  |
|  | ACTTTCAATCCCTTATGGGATTCTTC |  |
|  | CTTTCAATCCCTTTTGGGATTCATC |  |
| Sulfolobus solfataricus | GATTAATCCCAAAAGGAATTGAAAG | J |
|  | CTTTCAATTCCTTTTGGGATTAATC | g |
|  | CTTTCAATTCTATAAGAGATTATC | G |
|  | GATAATCTCTTATAGAATTGAAAG | J |
|  | AGATAATCTACTATAGAATTGAAAG | j |
| Sulfolobus tokodaii | CTTTCAATTCCTTTTGGGATTCATC |  |
|  | GATGAATCCCAAAAGGAATTGAAAG |  |
|  | CTTTCAATTCCATTAAGGATTATC |  |
|  | aagagCTTTCAATTCCATTAAGGATTATC |  |
| Symbiobacterium thermophilum IAM14863 | GTAGCACCCGGCCGCGAGGCCGGGTGAGGATTGAAAC |  |
|  | GTCGTCCCCACGCGCGTGGGGGTGAACCG | G |
|  | CGCGCAGAACCTGAAGTG | FP |
|  | CGCGCAAAACCTGAACCGCG | FP |
| Synechocystis PCC6803 (JZ) | CTTTCCTTCTACTAATCCCGGCGATCGGGACTGAAAC |  |
|  | GTTCAACACCCTCTTTTCCCCGTCAGGGGACTGAAAC |  |
|  | GTCTCCACTCGTAGGAGAAATTAATTGATTGGAAAC |  |
| Syntrophus aciditrophicus SB | GACAGAATTGACCTGATTTACGAAGGGATTGCGAC |  |
|  | GTTTCAATCCACGCCCCCGCATGGGGGGCGAC |  |
| Thermoanaerobacter tengcongensis | GTTTTTAGCCTACCTAAAAGGGATTGAAAC |  |
|  | GTTTCAATCCCTCTTAGGTAGGCTAAAAAC | G |
| Thermobifida fusca YX | GGATCATCCCCGCGTGCGCGGGGAGCACA |  |
|  | GTGCTCCCCACGCACGTGGGGATGGTCC |  |
|  | GGTCCATCCCCACGTGCGTGGGGAGCAT |  |
|  | GGTCCATCCCCACGTGCGTGGGGCTCAC |  |
|  | CGGTCCATCCCCACGTGCGTGGGGCTCAC |  |
|  | GAGAGCCCCACGCACGTGGGGATGGACCG |  |
| Thermococcus kodakaraensis KOD1 | TTTCAATTCTCCTAGAGTCTTATTGCAAC |  |
|  | GTTGCAATAAGACTCTAAGAGAATTGAAA |  |
| Thermoplasma acidophilum (JZ) | GTAAAATAGAACCTTAATAGGATTGAAAG |  |
| Thermoplasma volcanium | CTTCCATACTAACTAGTACATCTTAAAC | J |
| Thermotoga maritima | GTTTCAATACTTCCTTAGAGGTATGGAAAC | J |
|  | GTTTCCATACCTCTAAGGAATTATTGAAAC | G J |
| Thermus thermophilus HB27 (J-) | catacGTTGCAAGGGATTGAGCCCCGTAAGGGGATTGCgac | Q |
|  | GTCGCAATCCCCTTACGGGGCTCAATCCCTTGCAAC |  |
|  | GTTGCAAGGGATTGAGCCCCGTAAGGGGATTGCGAC |  |
|  | GTTTCAATCCTCACCGGCCCTTTCGGGCCGGTGCAAC |  |
|  | GTTGCACCGGCCCGAAAGGGCCGGTGAGGATTGAAA |  |
|  | GTTTCAATCCTCTACGAGGCTAACGAGGTTTGCAAC |  |
|  | GTTGCAAACCTCGTTAGCCTCGTAGAGGATTGAAAC |  |
| Thermus thermophilus HB8 | GTTGCAAGGGATTGAGCCCCGTAAGGGGATTGCGAC |  |
|  | GTCGCAATCCCCTTACGGGGCTCAATCCCTTGCAACcc |  |
|  | CGGTCCATCCCCACGTGCGTGGGGACTAC |  |
|  | GTAGTCCCCACGCGTGTGGGGATGGACCG |  |
|  | GTTTCAATCCTCTACGAGGCTGACGGGGTTTGCAAC | G |
| Treponema denticola ATCC 35405 | GTTTGAGAGTTGTGTAATTTAAGATGGATCTCAAAC |  |
| Vibrio vulnificus YJ016 | GTTTCAGACATGCCCGGTTTAGACGGGATTAAGAC | G |
| Wolinella succinogenes | GTTATAGCCGCCTACTCAGCCATTCCTCGCTATAAT | G |
|  | GCAACACTTTATAGCAAATCCGCTTAGCCTGTGAAAC | G |
| Xanthomonas axonopodis | TGGAAACGGTCAACGGC | Q |
|  | GTCGCGCCCTCACGGGCGCGTGGATTGAAAC | G |
| Xanthomonas citri | TGGAAACGGTCAACGGC | Q |
|  | GTCGCGCCCTCACGGGCGCGTGGATTGAAAC |  |
| Xanthomonas oryzae KACC10331 | GTTTCAATCCACGCGCCCGTGAGGACGCGAC | G |
| Yersinia pestis biovar Mediaevails | TTTCTAAGCTGCCTGTGCGGCAGTGAAC |  |
|  | aTTTCTAAGCTGCCTGTGCGGCAGTGAACtcg |  |
| Yersinia pestis CO92 | GTTCACTGCCGCACAGGCAGCTTAGAAA | g |
|  | TTTCTAAGCTGCCTGTGCGGCAGTGAAC |  |
| Yersinia pestis KIM | GTTCACTGCCGCACAGGCAGCTTAGAAA |  |
|  | ATTTTCTAAGCTGCCTGTGCGGCAGTGAACCT |  |
| Yersinia pseudotuberculosis IP32953 | GTTCACTGCCGCACAGGCAGCTTAGAAA | G |
|  | TTTCTAAGCTGCCTGTGCGGCAGTGAAC |  |
| Zymomonas mobilis ZM4 | TTTCTAAGCTGCCTATGCGGCAGTGAAC |  |
|  | GTTCACTGCCGCACAGGCAGCTTAGAAA | G |

### Supplementary Table 2

| Actinobacillus pleuropneunoniiae |
| --- |
| Agrobacterium tumefaciens C58 Cereon |
| Agrobacterium tumefaciens C58 UWash |
| Anaeromyxobacter dehalogenans 2CP-C |
| Anaplasma marginale St Maries |
| Anaplasma phagocytophilum HZ |
| Aster yellows witches-broom phytoplasma AYWB |
| Bacillus anthracis A2012 |
| Bacillus anthracis Ames |
| Bacillus anthracis Ames 0581 |
| Bacillus anthracis str Sterne |
| Bacillus cereus ATCC 10987 |
| Bacillus cereus ZK |
| Bacillus licheniformis ATCC 14580 |
| Bacillus licheniformis DSM 13 |
| Bacillus subtilis |
| Bacillus thuringiensis konkukian |
| Bacteroides thetaiotaomicron VPI-5482 |
| Bartonella henselae Houston-1 |
| Bartonella quintana Toulouse |
| Bdellovibrio bacteriovorus |
| Bifidobacterium longum |
| Bordetella avium 197N |
| Bordetella bronchiseptica |
| Bordetella parapertussis |
| Bordetella pertussis |
| Borrelia burgdorferi |
| Borrelia garinii PBi |
| Bradyrhizobium japonicum |
| Brucella abortus 9-941 |
| Brucella melitensis |
| Brucella melitensis biovar Abortus |
| Brucella suis 1330 |
| Buchnera aphidicola |
| Buchnera aphidicola Sg |
| Buchnera sp |
| Burkholderia mallei ATCC 23344 |
| Burkholderia pseudomallei 1710b |
| Burkholderia pseudomallei K96243 |
| Burkholderia thailandensis E264 |
| Burkholderia xenovorans LB400 |
| Candidatus Blochmannia floridanus |
| Candidatus Blochmannia pennsylvanicus BPEN |
| Candidatus Pelagibacter ubique HTCC1062 |
| Caulobacter crescentus |
| Chlamydia muridarum |
| Chlamydia trachomatis |
| Chlamydia trachomatis A HAR-13 |
| Chlamydophila abortus S26 3 |
| Chlamydophila caviae |
| Chlamydophila felis Fe C-56 |
| Chlamydophila pneumoniae AR39 |
| Chlamydophila pneumoniae CWL029 |
| Chlamydophila pneumoniae J138 |
| Chlamydophila pneumoniae TW 183 |
| Clostridium acetobutylicum |
| Clostridium perfringens |
| Colwellia psychrerythraea 34H |
| Corynebacterium glutamicum ATCC 13032 Bielefeld |
| Corynebacterium glutamicum ATCC 13032 Kitasato |
| Coxiella burnetii |
| Dechloromonas aromatica RCB |
| Dehalococcoides ethenogenes 195 |
| Deinococcus radiodurans |
| Ehrlichia canis Jake |
| Ehrlichia chaffeensis Arkansas |
| Ehrlichia ruminantium Gardel |
| Ehrlichia ruminantium str. Welgevonden |
| Ehrlichia ruminantium Welgevonden |
| Enterococcus faecalis V583 |
| Erythrobacter litoralis HTCC2594 |
| Escherichia coli CFT073 |
| Francisella tularensis holarctica |
| Francisella tularensis tularensis |
| Gloeobacter violaceus |
| Gluconobacter oxydans 621H |
| Haemophilus ducreyi 35000HP |
| Haemophilus influenzae |
| Haemophilus influenzae 86 028NP |
| Halobacterium sp |
| Helicobacter hepaticus |
| Helicobacter pylori 26695 |
| Helicobacter pylori J99 |
| Idiomarina loihiensis L2TR |
| Jannaschia CCS1 |
| Lactobacillus johnsonii NCC 533 |
| Lactobacillus plantarum |
| Lactobacillus sakei 23K |
| Lactococcus lactis |
| Legionella pneumophila Paris |
| Legionella pneumophila Philadelphia 1 |
| Leifsonia xyli xyli CTCB0 |
| Listeria monocytogenes 4b F2365 |
| Magnetospirillum magneticum AMB-1 |
| Mesoplasma florum L1 |
| Mesorhizobium loti |
| Methanococcus maripaludis S2 |
| Mycobacterium avium paratuberculosis |
| Mycobacterium leprae |
| Mycoplasma genitalium |
| Mycoplasma hyopneumoniae 232 |
| Mycoplasma hyopneumoniae 7448 |
| Mycoplasma hyopneumoniae J |
| Mycoplasma mycoides |
| Mycoplasma penetrans |
| Mycoplasma pneumoniae |
| Mycoplasma pulmonis |
| Neisseria gonorrhoeae FA 1090 |
| Neisseria meningitidis MC58 |
| Neorickettsia sennetsu Miyayama |
| Nitrobacter hamburgensis X14 |
| Nostoc PCC7120 |
| Novosphingobium aromaticivorans DSM 12444 |
| Oceanobacillus iheyensis |
| Onion yellows phytoplasma |
| Pelobacter carbinolicus |
| Pirellula sp |
| Polaromonas JS666 |
| Prochlorococcus marinus CCMP1375 |
| Prochlorococcus marinus MED4 |
| Prochlorococcus marinus MIT 9312 |
| Prochlorococcus marinus MIT9313 |
| Prochlorococcus marinus NATL2A |
| Propionibacterium acnes KPA171202 |
| Pseudoalteromonas haloplanktis TAC125 |
| Pseudomonas aeruginosa |
| Pseudomonas fluorescens Pf-5 |
| Pseudomonas fluorescens PfO-1 |
| Pseudomonas putida KT2440 |
| Pseudomonas syringae |
| Pseudomonas syringae phaseolicola 1448A |
| Pseudomonas syringae tomato |
| Psychrobacter arcticum 273-4 |
| Ralstonia eutropha JMP134 |
| Ralstonia solanacearum |
| Rhizobium etli CFN 42 |
| Rhodobacter sphaeroides 2 4 1 |
| Rhodopseudomonas palustris CGA009 |
| Rhodopseudomonas palustris HaA2 |
| Rickettsia bellii RML369-C |
| Rickettsia conorii |
| Rickettsia prowazekii |
| Rickettsia typhi wilmington |
| Saccharophagus degradans 2-40 |
| Salinibacter ruber DSM 13855 |
| Shewanella denitrificans OS217 |
| Shewanella oneidensis |
| Shigella boydii Sb227 |
| Shigella dysenteriae |
| Shigella flexneri 2a |
| Shigella flexneri 2a 2457T |
| Shigella sonnei Ss046 |
| Silicibacter pomeroyi DSS-3 |
| Sinorhizobium meliloti |
| Sodalis glossinidius morsitans |
| Staphylococcus aureus aureus MRSA252 |
| Staphylococcus aureus aureus MSSA476 |
| Staphylococcus aureus COL |
| Staphylococcus aureus Mu50 |
| Staphylococcus aureus MW2 |
| Staphylococcus aureus N315 |
| Staphylococcus aureus NCTC 8325 |
| Staphylococcus aureus RF122 |
| Staphylococcus aureus USA300 |
| Staphylococcus epidermidis ATCC 12228 |
| Staphylococcus haemolyticus |
| Staphylococcus saprophyticus |
| Streptococcus pneumoniae R6 |
| Streptococcus pneumoniae TIGR4 |
| Streptococcus pyogenes MGAS10394 |
| Streptococcus pyogenes MGAS315 |
| Streptococcus pyogenes MGAS8232 |
| Streptococcus pyogenes SSI-1 |
| Streptomyces coelicolor |
| Synechococcus CC9605 |
| Synechococcus CC9902 |
| Synechococcus elongatus PCC 6301 |
| Synechococcus elongatus PCC 7942 |
| Synechococcus sp WH8102 |
| Thermosynechococcus elongatus |
| Thiobacillus denitrificans ATCC 25259 |
| Thiomicrospira crunogena XCL-2 |
| Thiomicrospira denitrificans ATCC 33889 |
| Treponema pallidum |
| Tropheryma whipplei TW08 27 |
| Tropheryma whipplei Twist |
| Ureaplasma urealyticum |
| Vibrio cholerae |
| Vibrio fischeri ES114 |
| Vibrio parahaemolyticus |
| Vibrio vulnificus CMCP6 |
| Wigglesworthia brevipalpis |
| Wolbachia endosymbiont of Brugia malayi TRS |
| Wolbachia endosymbiont of Drosophila melanogaster |
| Xanthomonas campestris |
| Xanthomonas campestris 8004 |
| Xanthomonas campestris vesicatoria 85-10 |
| Xylella fastidiosa |
| Xylella fastidiosa Temecula1 |

### Supplemental Table 1.

**Putative CRISPR repeats reported by PILER-CR.**

PILER-CR was run on 346 prokaryotic genomes. This table shows all predicted repeats, with redundancy reduced by eliminating sequences that are ≥ 90% identical to another reported sequence within the same species. Those genomes for which no repeats were reported are listed in Table 3. The *Notes* column contains the following codes: FP=probable false positive, Q=questionable prediction, G=exact agreement with Godde and Bickerton sequence, g=close match with Godde and Bickerton sequence, J=exact match with Jensen *et al.* sequence, j=close match with Jensen *et al*. sequence, J+=sequence found by PILER-CR but not reported by Jensen *et al*. Species marked JZ were reported to have no CRISPR repeats by Jensen *et al.* JZ and J+ may therefore be interpreted as cases where PILER-CR shows improved sensitivity over the methods of Jensen *et al.* Lower-case letters indicate cases where manual inspection suggested that these letters should be deleted from the consensus repeat; similarly, underlined upper-case letters are cases where manual inspection suggested that these letters should be added to the sequence. The notation {GT,C} indicates a case where two arrays with very similar consensus sequences were concatenated by PILER-CR. One array has consensus sequence GT*s*, where *s* is some sequence, and the other has consensus sequence C*s*. (Or *s*GT and *s*C, if the end of the consensus varies). In such cases it may not be clear whether the array should be split into two.

### Supplemental Table 2.

**Species for which PILER-CR reported no CRISPR repeats.**

Here we provide a list of those species for which no putative arrays were reported. In just one case, *Mycobacterium avium*, a repeat was reported in a previous study (Jensen *et al.*). As we were unable to find the previously reported repeat in *Mycobacterium avium paratuberculosis*, we assume that the discrepancy is explained by the use of different strains.
